# Status Quo of Feline Leukaemia Virus Infection in Turkish Cats and Their Antigenic Prevalence

**DOI:** 10.3390/ani14030385

**Published:** 2024-01-25

**Authors:** Emrah Korkulu, Elif İrem Şenlik, Ece Adıgüzel, Fatma Gökçe Artut, Hüseyin Doğukan Çetinaslan, Eda Erdem-Şahinkesen, Tuba Çiğdem Oğuzoğlu

**Affiliations:** 1 Institute of Health Sciences, Ankara University, Ankara 06110, Türkiye; korkuluemrah@icloud.com (E.K.); elifsenlikirem@gmail.com (E.İ.Ş.); dcetinaslan@gmail.com (H.D.Ç.); 2 Republic of Türkiye Ministry of Agriculture and Forestry, Atkaracalar District Directorate, Çankırı 18310, Türkiye; adiguzelece@gmail.com; 3 Cankoru Animal Hospital, Ankara 06810, Türkiye; gokce_1303_@hotmail.com; 4 Department of Virology, Faculty of Veterinary Medicine, Ankara University, Ankara 06110, Türkiye

**Keywords:** FeLV, antigenic prevalence, Türkiye

## Abstract

**Simple Summary:**

Feline leukaemia virus (FeLV) is a significant infectious agent in cats that is pre-valent worldwide. This study aims to evaluate the current situation in Turkish cats, compare them with other strains in the world via molecular characterization, and evaluate other parameters in cats with this infection.

**Abstract:**

Feline leukaemia virus (FeLV) is a member of the Gammaretrovirus genus, which has two genotypes in cats: endogenous (replication-defective provirus) and exogenous (replication-competent). In this study, 550 cats were examined, and 112 of them (20.36%) were found to have the endogenous FeLV (enFeLV) genotype. EnFeLV-positive animals were also tested for additional viral infections, and 48 cats (42.85%) were discovered to be co-infected with other viruses. According to co-infection data, these cats were infected with feline coronavirus (FCoV) (27/112, 24.1%), feline panleukopenia virus (FPV) (14/112, 12.5%), feline immunodeficiency virus (FIV) (0/112, 0%), and domestic cat hepadnavirus (DCH) (13/112, 11.6%). Their age, sex, breed, clinical state, lifestyle (in/outdoor), and immunization data against FeLV were also evaluated. In line with our results, the prevalence of enFeLV and co-infection with other pathogens in cats admitted to the clinic for various reasons were discussed. The majority of positive animals in terms of FeLV (94/112, 83.93%) had clinical findings. We emphasized that the FeLV-positive situation of cats should be taken into consideration by veterinarians when planning treatment and vaccination programs. Additionally, in this study, we questioned the group in which our enFeLVs were phylogenetically located. Therefore, we performed a phylogenetic analysis based on a comparison with global FeLV sequences obtained from the GenBank database. The sequenced positive samples were in the AGTT subgroup within Group-II.

## 1. Introduction

Feline leukemia virus (FeLV) is a *Gammaretrovirus* that infects both domestic, and non-domestic felids [1,2]. FeLV is thought to have originated from a rodent-borne virus that infected cats due to the predator/prey relationship between cats and mice [3]. FeLV is one of the most important pathogenic viruses in cats, predominantly causing hematolo-gical diseases ranging from non-proliferative to proliferative with immunosuppression [4]. FeLV is classified into two genotypes: exogenous (exFeLV) and endogenous (enFeLV) [5]. According to reports, the exFeLV genotype is horizontally transmitted by close contact, mutual grooming, wounds, and bites [6], and the enFeLV genotype is transmitted by vertical transmission [7].

According to Benveniste and Todaro, the endogenous type of retroviruses in *Felidae* is a replication-defective provirus that can integrate into germ line cells or arise during early embryogenesis, and enFeLV sequences are found in 6–12 copies per genome [8]. Furthermore, several FeLV-associated endogenous elements with intact long terminal repeats (LTRs) may be found in the genome of domestic cats [9,10], many of which are polymorphic [11]. Although these defective enFeLVs cannot produce infectious virions, they may occur in recombination with exFeLVs [12]. This recombination may result in the formation of novel variants, which may lead to severe pathogenesis [13].

Six FeLV subgroups have been identified to date (FeLV-A, FeLV-B, FeLV-C, FeLV-D, FeLV-E, FeLV-T) [14,15]. The major subgroup lacking enFeLV elements is FeLV-A. FeLV-B, -C, -D and -E occurs via a recombination of enFeLV and exFeLV subgroup A, and these subgroups have affinity for B lymphocytes [15,16,17]. The FeLV-T subgroup is known to arise because of mutations in FeLV-A and a T cell trophic cytopathic virus, which causes lymphoid depletion and immunodeficiency in infected cats [18,19].

In genomic studies of FeLVs, the U3 regions of the LTRs of enFeLVs and exFeLVs show no homology. Therefore, to distinguish these two genotypes, the sequences of this region are used in hybridisation studies. Phylogenetically, enFeLVs are classified into Group I and Group II, while exFeLVs are not categorised. In several studies, Group II is further divided into two monophyletic groups in domestic cats: AGTT and AGTT-like. These four bases originate from the host genome. Studies on enFeLV LTRs found in domestic cats suggest that the formation of the two aforementioned groups in these species is possibly due to genome invasions that occurred at two different times in their ancestors. With this, it is hypothesised that, throughout their evolutionary history, domestic cat genomes may have been targeted by FeLVs more than once. Therefore, it is considered possible that one of these two groups originated before the other. Furthermore, the frequency of AGTT observed in domestic cats but not in wild cats suggests that genome integration occurred after, or simultaneously with, the domestication of cats [16,20].

This study was planned due to the increase in FeLV positivity observed in our recent routine diagnostic examinations. EnFeLV positivity was found in all cats investigated in this study. In addition to this, we evaluated noted individual features, such as age, sex, breed, lifestyle, clinical parameters, vaccination status against FeLV, and viral co-infections, and their role in the increase in FeLV infection among cats.

## 2. Materials and Methods

### 2.1. Animals and Sampling

Blood samples of 550 domestic cats were obtained from private veterinary clinics and hospitals in Ankara. These samples were collected with an informed consent form signed by the cat owners, following the direction of the Ankara University Local Ethics Committee for Animal Experimentation (2020/13/111). The cats differed in terms of their clinical symptoms, sex, breed, age, living conditions, and vaccination status against FeLV (Table 1).

### 2.2. Diagnosis of Viral Nucleic Acids by Polymerase Chain Reaction (PCR)

The conventional extraction method (phenol/chloroform/isoamyl alcohol, 25:24:1) was used for the viral nucleic acid’s isolation [21]. The primer pairs, based on env-LTR, as reported by Polani et al., were used for enFeLV identification via conventional PCR [20]. For exFeLV detection, the primer pair for the U3-LTR region described by Herring et al. was used [22]. Additionally, all samples were investigated for various systemic viral infections—feline coronavirus (FCoV), feline panleukopenia virus (FPV), feline immunodeficiency virus (FIV), and domestic cat hepadnavirus (DCH)—using previously described PCR techniques [23,24,25,26]. PCR products were visualized on a UV transilluminator (Gel Logic 100, Kodak, Rochester, NY, USA) via electrophoresis on 1% agarose gels containing SafeView™ DNA Stains (Applied Biological Materials, Richmond, BC, Canada).

### 2.3. Sequencing of FeLV-Positive PCR Amplicons and Their Phylogenetic Analysis

Nine of the PCR amplicons were selected and purified using a High Pure PCR Cleanup Micro Kit (Roche, Basel, Switzerland). Our sample selection criteria for sequence analysis were the presence of mixed infections and multiple clinical symptoms. Selected samples were subjected to sequencing using the Sanger method. The sequences were compared with references in the GenBank database in BLAST program. The sequence data were aligned using BioEdit (version 7.1.3.0) [27]. A phylogenetic tree was created using MEGA X software (version 10.0.1) [28] according to the maximum likelihood, Kimura-2 parameter bootstrap 1000 method, according to the classification criteria of Polani et al. and Roca et al. [16,20].

### 2.4. Statistical Analysis on FeLV-Positive Cats

The information on FeLV-positive cats obtained in this study was statistically analysed. Chi-square analysis was used for statistical comparisons between groups according to sex, health, lifestyle, and vaccination status against FeLV in SPSS (SPSS v19.0, Chicago, IL, USA). As a result of the analysis, those with a probability value less than 0.05 were considered statistically significant (*p* < 0.05).

## 3. Results

In this study, the prevalence rate of enFeLV was found to be 20.36% (112/550) in the sampled cats, and no exogenous FeLV positivity was detected. The 112 enFeLV-positive cats were investigated for other systemic viral infections, including FPV, FCoV, DCH, and FIV. Of the FeLV-positive cats, 57.1% (64/112) were infected with only enFeLV and 42.9% (48/112) were co-infected with other viruses. The positivity rates of co-infections in enFeLV-infected cats were as follows: FCoV, 19.6% (22/112); FPV, 8% (9/112); FCoV + FPV, 3.6% (4/112); DCH, 9.8% (11/112); DCH + FCoV, 0.9% (1/112); and DCH + FPV, 0.9% (1/112). No positivity was found for FIV infection. The results of all tests for viral infections are summarised in Table 1.

The rate of sick male cats showing clinical symptoms (93.2%) was significantly higher than the rate of sick female cats (73.6%) (*p* < 0.01). The rate of those cats showing clinical signs in cats living outdoors (97.7%) was found to be significantly higher than that of in cats living indoors and showing clinical signs (74.6%) (*p* < 0.001) (Table 2).

While the prevalence of only FeLV was 60% in those who were not vaccinated against FeLV, the prevalence was 69% in those who were vaccinated. No significant difference was detected between these values (*p* > 0.05) (Table 3).

Regarding health status, clinical signs and symptoms showed a significant relationship with disease occurrence (*p*< 0.05). A similar relationship was also seen between sex and life status of enFeLV-positive cats. Cats aged 7–48 months were found to be mostly positive for enFeLV in this study. However, age was not found to be a risk factor for the presence of enFeLV.

**Figure 1:**
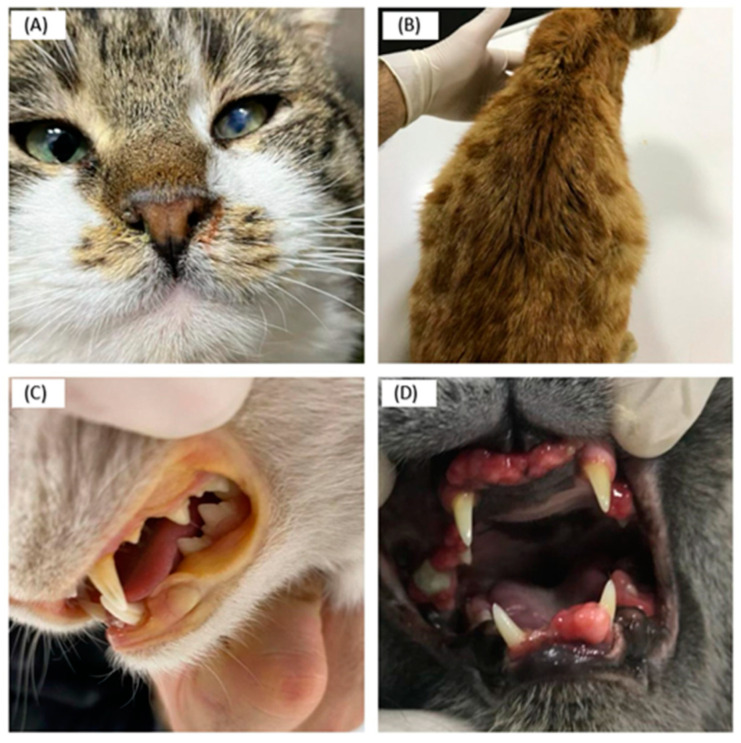
(**A**) Clinical symptoms of a detected case of enFeLV: uveitis in the left eye. (**B**) Clinical symptoms of a detected case of FCoV and enFeLV: Ascites. (**C**) Clinical symptoms of a detected case of enFeLV: icterus in the oral mucosa. (**D**) Clinical symptoms of a detected case of enFeLV: gingival hyperplasia.

Regarding the concept of “health status”, the entire population comprised 18 healthy and 94 sick cats based on clinical diagnosis. Namely, of the 112 cats found to be positive for enFeLV, 83.93% (94/112) showed various clinical symptoms, while 16% (18/112) had no clinical symptoms. The clinical symptoms of sick cats included haematological disorders (leukopenia, anaemia, icterus), general disorders (lethargy, anorexia, fever, lymphadenopathy), gingivitis/stomatitis, incoordination, uveitis, ascites/pleural effusion, respiratory diseases, skin diseases, and gastrointestinal and urogenital diseases. The most common clinical finding in cats infected only with enFeLV was anorexia, followed by gingivitis and stomatitis (Table 4 and Figure 1).

**Figure 2:**
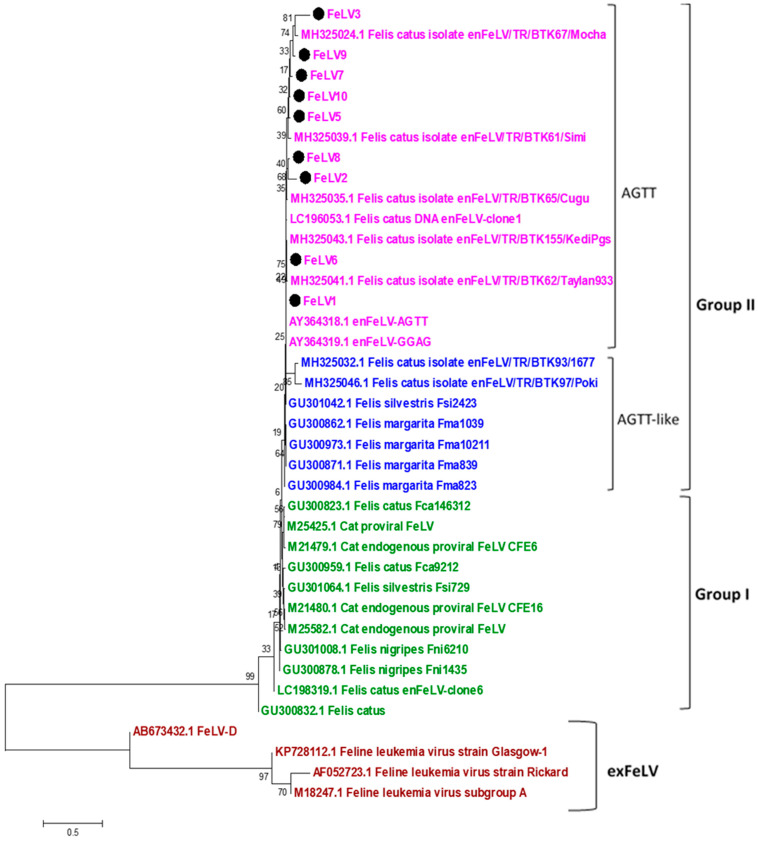
The phylogenetic tree based on the env-LTR gene region was created using the maximum likelihood method based on the Kimura 2-parameter model. Evolutionary analyses were conducted using MEGA X. In the classification made by Roca et al. using these gene regions, enFeLVs were divided into two subgroups: Group I and Group I. There was no categorization among exFeLVs. The sequences included in this study (labelled) were classified as AGTT in group II.

**Figure 3:**
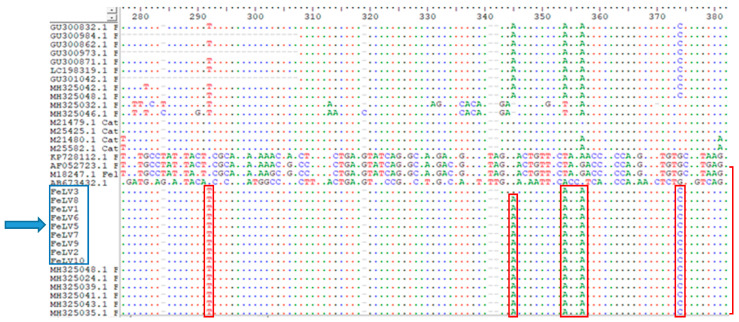
Nucleic acid similarities in Turkish strains are marked in red. Nine sequences from this study, indicated by arrows, were in the AGTT subgroup within Group II.

Sequence analysis was performed on 9 samples with mixed infection out of the 112 enFeLV-positive samples. The sequences were compared with themselves, with other enFeLV groups from around the world, and with exFeLV sequences. According to the phylogenetic classification, the samples were determined to be in the AGTT-monophyletic group within Group II (Figure 2 and Figure 3).

## 4. Discussion

In recent years, we increasingly detected FeLV nucleic acid positivity in cats (20.36%, 112/550 in this study). This increase was also noted in a previous comprehensive study conducted in Türkiye [29]; however, in the current study, the increase in FeLV positivity was even greater than that in the previous study. The present study aimed to determine the current status of enFeLV positivity to draw veterinarians’ attention to this infection, as well as to examine the enFeLVs in Türkiye from a phylogenetic perspective. In addition, we questioned whether sequence variations from the monophyletic branch were mentioned in the data.

Phylogenetic studies on enFeLV are limited. The first study on the classification of enFeLVs was performed by Roca et al., reporting that enFeLVs can be divided into two groups [16]. Group I was not separated into branches. However, Group II was divided into two separate branches. These branches were named according to the four bases they derive from the host genome, which are AGTT and AGTT-like. The enFeLV sequences obtained in this study belonged to the same group as the enFeLV AGTT strain of American origin in Group II, as revealed by the phylogenetic analysis with reference to other enFeLV sequences worldwide. Although the impact of monophyletic branching differences on pathogenesis is unknown, this phylogenetic differentiation is thought to be related to the co-evolution of cats and endogenous FeLV elements. Thus, the identification of enFeLV species in domestic cats provides information about the evolutionary history of domestic cats [16,20]. A recent study on FeLV in Türkiye [29] reported the presence of Group II enFeLV, aligning with our findings. However, the increase in the prevalence of enFeLV in Türkiye was remarkable.

Of the enFeLV-positive cats in this study, 83.9% (94/112) showed clinical signs and symptoms, and 16.1% (18/112) were healthy. In a study conducted in Rio de Janeiro, the prevalence of FeLV was found to be 11.52% (126/1094). It was reported 70.63% (89/126) of positive cats had clinical findings [30]. In our study, a wide range of clinical signs were observed in the 94 symptomatic FeLV-infected cats, mainly anorexia, lethargy, gingivitis/stomatitis, and incoordination. Our findings are consistent with those of Studer et al. [31]. The healthy cats in the current study were brought to the veterinary clinic for routine procedures such as vaccination, castration, or check-ups. The distribution of enFeLV in sick cats with various clinical signs and symptoms is high. Although this accumulation was found to be statistically significant (*p* < 0.05), the appearance of these symptoms may be related to the presence of other infections. In addition, 51% (48/94) of the cats with clinical signs and symptoms in our study were also found to be co-infected with other viral pathogens (FCoV, FPV, DCH) that cause systemic disease and are encountered in veterinary clinics. While enFeLV infection alone does not usually cause clinical disease [32]. The presence of this factor in cats is considered as the underlying triggering agent, along with other pathogens. This situation should be considered by veterinary clinicians.

Even if enFeLV infection alone generally does not cause serious disease, its ability to cause immunosuppression can trigger other infections. In support of this statement, previous studies have emphasised that the presence of FeLV in FCoV-infected cats predisposes them to the formation of FIP [33]. Considering the immunosuppressive properties of this virus, we assessed the co-existence of other viral agents (FCoV, FPV, FIV and DCH) with enFeLV, which cause the systemic diseases frequently encountered by veterinarians. Co-infection was detected in 42.85% (48/112) of the cats in our study, and 56.25% (27/48) of these cats were found to be infected with FCoV. These results are compatible with the extant literature on the association between FeLV and FCoV [33]. FPV and DCH dual-co-infection rates were also found to be significant. In the presence of FeLV infection, either experimental infections or long-term studies of closed populations are needed to evaluate the emergence of other viral agents. Since our study used samples from a single time point, it was not possible to evaluate interactions between diseases; however, the presence of multiple infections in the same individual was determined.

A study [33] of a large colony of domestic cats living in a closed environment reported on the viral pathogenicity and co-infection interactions that occurred during a natural FeLV infection outbreak with high morbidity. In our study, a total of six animals with triple infection were identified. Four cats were infected with FCoV, FPV, and enFeLV. In one cat, both FCoV and DCH were detected, and in another, both FPV and DCH were found, in addition to enFeLV.

In this study, most of the enFeLV-positive cats were Tabby cats. The low *p*-value (*p* < 0.05) associated with the breed in enFeLV-positive cats and the fact that most positive cats were of the Tabby breed is probably due to the fact that the cat population in Türkiye is predominantly of this breed. In this study, all the outdoor cats and the majority of indoor cats that were adopted from the street were of the Tabby breed. For this reason, we believe that the breed effect on FeLV infection in cats in our country should be clarified in future studies.

When the effect of lifestyle on enFeLV positivity was questioned, no significant difference (*p* > 0.05) was observed between the two groups (indoor/outdoor). On the other hand, clinical findings were present in 97.7% (42/43) of cats living outdoors and in 74.6% (50/67) of cats living indoors. The relationship between lifestyle and health status was evaluated statistically; this difference was found to be significant (*p* < 0.001).

Although age has not been implicated as a risk factor for FeLV infection, factors such as sexual maturity, pregnancy, stress due to changes in living conditions, and the presence of other infections should be taken into account when considering enFeLV in cats. Since most of the positive animals were older than 7 months in this study and the distribution was non-equal between groups, it was not possible to establish a statistically significant relationship between age and FeLV infection (*p* > 0.05). On the other hand, detecting enFeLV positivity in cats that are 0–6 months old could be evidence the presence of vertical transmission.

We found enFeLV positivity in 59 male and 53 female cats. No significant difference (*p* > 0.05) was observed in terms of sex. These results are compatible with the findings of Koç and Oğuzoğlu and Powers et al. [29,33]. A total of 93.2% (55/59) of male cats and 73.6% (39/53) of female cats presented with clinical signs and symptoms. This high rate of clinical findings in male cats was found to be statistically significant (*p* < 0.01). Although previous studies have questioned whether factors such as age, sex, and breed may be important in the transmission of FeLV infection, definitive evidence for this hypothesis has not been reported to date. The findings of our study are similar.

The main purpose of vaccines is to protect cats by inducing antibodies against diseases before they encounter infectious agents. Although the immune-boosting effect of colostrum varies from cat to cat, kittens receiving colostrum are generally protected against diseases for a period of from 8 to 18 weeks. However, since they may be vulnerable to infectious diseases after this period, it is important to plan appropriate vaccination programmes. Vaccination programmes for cats in Türkiye are usually completed by 6 months of age. Such programmes include several viral vaccines (Feline Calicivirus, Feline Herpesvirus and Feline Panleukopenia virus, FeLV and rabies virus). Anamnesis information and hemogram results are usually evaluated prior to beginning the vaccination programme. In the vaccination guide prepared by The World Small Animal Veterinary Association (WSAVA) Vaccination Guidelines Group (VGG), vaccines for pets are divided into core and non-core types. Core vaccines are recommended for all kittens and adult cats. They are applied to provide protection against the most common life-threatening infectious diseases. Non-core vaccines do not need to be administered to every cat. The FeLV vaccine is in the “non-core” group [34]. In Türkiye, FeLV vaccines are part of routine vaccination programmes. However, the presence of FeLV antigenemia is generally disregarded. Studies on enFeLV are reported to be more common in wild and domestic felines than those on exFeLV. Additionally, FeLV vaccines have been declared not to prevent proviral integration [18,35]. Of the 112 cats in our study, 42 were vaccinated against FeLV, 20 were unvaccinated, and information on 50 could not be obtained. All the vaccinated cats were indoor cats. While the prevalence of only FeLV was 60% (12/20) in those who were not vaccinated against FeLV, the prevalence was 69% (29/42) in those who were vaccinated, and no significant difference was detected between these values (*p* > 0.05). The results of this study show that the presence of FeLV infection should be questioned by veterinarians. In our opinion, the evaluation of hemogram results, along with a clinical examination and antigenic investigation of FeLV infection before vaccination, is very important. It should not be forgotten that vaccines that are used to combat infections should be administered to at-risk individuals for protection purposes.

## 5. Conclusions

The data that have been obtained in recent years about FeLV infection in Türkiye show that this infection is gradually increasing. In this context in this study, we drew attention to the rising prevalence of enFeLV positivity in Türkiye. Our findings revealed that Turkish enFeLV strains belong to Group II. Exogenous FeLV positivity has not been reported in Türkiye to date, including in this study. We assessed the presence of this infection in cats while also considering other factors (age, sex, breed, clinical state, lifestyle, and immunization data against FeLV). It was determined that there was a statistically significant increase in clinical findings in male cats and outdoor animals. We also questioned whether this infection, which has an immunosuppressive nature, was associated with other viral infections. We determined that FCoV > FPV > DCH infections were common in cats with FeLV positivity. Given the increasing number of recent reports of FeLV variants that may increase the likelihood of recombination, it is important to consider this issue from both a cat health and disease interactions perspective. Our recommendation is that FeLV antigenemia (enFeLV and exFeLV) be taken into account when including FeLV vaccines in vaccination programs. In accordance with the FeLV risk–benefit analysis, a controlled vaccination program for cats should be implemented in shelters with more than one household.

## Figures and Tables

**Table 1:** The number of FeLV, FCoV, FPV, and DCH positivity according to breed, sex, age, lifestyle, health status, and vaccination against FeLV.

		Total	Only FeLV	FeLV FCoV	FeLV FPV	FeLV FCoV FPV	FeLV DCH	FeLV DCH FCoV	FeLV DCH FPV
Breed	Tabby	74	35	16	7	4	10	1	1
	British	19	14	4	1	0	0	0	0
	Scottish	4	4	0	0	0	0	0	0
	Siamese	2	2	0	0	0	0	0	0
	Persian	2	0	2	0	0	0	0	0
	Mix	11	9	0	1	0	1	0	0
***p*** **-value < 0.05**									
Sex	Female	53	31	9	4	4	4	0	1
	Male	59	33	13	5	0	7	1	0
***p*** **-value > 0.05**									
Age (in months)	0–6	16	5	5	1	3	1	0	1
	7–12	36	20	4	5	1	5	1	0
	13–48	45	28	11	2	0	4	0	0
	>48	12	8	2	1	0	1	0	0
	Undetermined	3	3	0	0	0	0	0	0
***p*** **-value > 0.05**									
Lifestyle	Indoor	67	49	11	4	0	3	0	0
	Outdoor	43	13	11	5	4	8	1	1
	Undetermined	2	2	0	0	0	0	0	0
***p*** **-value > 0.05**									
Health status	Healthy	18	13	1	1	0	2	0	1
	Sick	94	51	21	8	4	9	1	0
***p*** **-value < 0.05**									
Vaccination *	Yes	42	29	10	3	0	0	0	0
	No	20	12	2	1	4	1	0	0
	Undetermined	50	23	10	5	0	10	1	1
***p*** **-value > 0.05**									
		112	64	22	9	4	11	1	1
**Percentages (%)**		100	57.1	19.6	8	3.6	9.8	0.9	0.9

* Vaccination against feline leukemia virus.

**Table 2:** Cross-tabulation of chi-square test evaluating sex, lifestyle and health status.

			Health Status		Total
			Sick	Healthy	
Sex *p* < 0.01	Female	Count	39	14	53
		% within sex	73.6%	26.4%	100.0%
	Male	Count	55	4	59
		% within sex	93.2%	6.8%	100.0%
Total		Count	94	18	112
		% within sex	83.9%	16.1%	100.0%
Lifestyle *p* < 0.001	Outdoor	Count	42	1	43
		% within lifestyle	97.7%	2.3%	100.0%
	Indoor	Count	50	17	67
		% within lifestyle	74.6%	25.4%	100.0%
Total		Count	92	18	110
		% within lifestyle	83.6%	16.4%	100.0%

**Table 3:** Cross-tabulation of chi-square test evaluating vaccine status against FeLV and positivity rates in terms of FeLV and others (FCoV, FPV, and DCH).

			Positivity		Total
			FeLV	FeLV and Others	
Vaccine *p* > 0.05	No	Count	12	8	20
		% within vaccine	60.0%	40.0%	100.0%
	Yes	Count	29	13	42
		% within vaccine	69.0%	31.0%	100.0%
Total		Count	41	21	62
		% within vaccine	66.1%	33.9%	100.0%

**Table 4:** Clinical and haematological findings of 94 sick cats.

	Total	Only FeLV	FeLV FCoV	FeLV FPV	FeLV FCoV FPV	FeLV DCH	FeLV DCH FCoV	FeLV DCH FPV
**Signs and symptoms**	94							
Leukopenia	22	10	4	3	4	1	0	0
Icterus	13	7	2	1	1	2	0	0
Anaemia	10	2	2	1	4	1	0	0
Ascites/pleural effusion	17	7	7	1	1	0	1	0
Uveitis	12	7	2	0	1	2	0	0
Stomatitis/Gingivitis	24	15	3	3	2	1	0	0
Rhinitis	11	5	5	1	0	0	0	0
Lymphadenopathy	16	6	5	1	3	1	0	0
Fever	11	5	1	2	2	1	0	0
Anorexia	50	23	16	5	4	2	0	0
Lethargy	34	14	9	5	4	2	0	0
Incoordination	17	11	2	2	0	2	0	0
Skin diseases	4	1	1	2	0	0	0	0
Respiratory diseases	9	4	1	2	1	1	0	0
Urogenital diseases	6	5	1	0	0	0	0	0
Gastrointestinal diseases	13	4	2	3	3	1	0	0

## Data Availability

The datasets presented in this study are publicly available.

## References

[1] Hoover E.A., Mullins J.I. (1991). Feline leukemia virus infection and diseases. J. Am. Vet. Med. Assoc..

[2] Miyazawa T. (2002). Infections of feline leukemia virus and feline immunodeficiency virus. Front. Biosci.-Landmark.

[3] Benveniste R.E., Sherr C.J., Todaro G.J. (1975). Evolution of type C viral genes: Origin of feline leukemia virus. Science.

[4] Stewart H., Jarrett O., Hosie M., Willett B. (2011). Are endogenous feline leukemia viruses really endogenous?. Vet. Immunol. Immunopathol..

[5] Anai Y., Ochi H., Watanabe S., Nakagawa S., Kawamura M., Gojobori T., Nishigaki K. (2012). Infectious endogenous retroviruses in cats and emergence of recombinant viruses. J. Virol..

[6] Hardy W.D., Hess P.W., MacEwen E.G., McClelland A.J., Zuckerman E.E., Essex M., Cotter S.M., Jarrett O. (1976). Biology of feline leukemia virus in the natural environment. Cancer Res..

[7] Krunic M., Ertl R., Hagen B., Sedlazeck F.J., Hofmann-Lehmann R., von Haeseler A., Klein D. (2015). Decreased expression of endogenous feline leukemia virus in cat lymphomas: A case control study. BMC Vet. Res..

[8] Benveniste R.E., Todaro G.J. (1975). Segregation of RD-114 and FeLV-related sequences in crosses between domestic cat and leopard cat. Nature.

[9] Soe L.H., Devi B.G., Mullins J., Roy-Burman P. (1983). Molecular cloning and characterization of endogenous feline leukemia virus sequences from a cat genomic library. J. Virol..

[10] Soe L.H., Shimizu R.W., Landolph J.R., Roy-Burman P. (1985). Molecular analysis of several classes of endogenous feline leukemia virus elements. J. Virol..

[11] Koshy R., Gallo R., Wong-Staal F. (1980). Characterization of the endogenous feline leukemia virus-related DNA sequences in cats and attempts to identify exogenous viral sequences in tissues of virus-negative leukemic animals. Virology.

[12] Roy-Burman P. (1995). Endogenousenv elements: Partners in generation of pathogenic feline leukemia viruses. Virus Genes.

[13] Eggers H.J. (2004). Principles of virology: Molecular biology pathogenesis and control of animal viruses. Int. J. Med. Microbiol..

[14] Lauring A.S., Anderson M.M., Overbaugh J. (2001). Specificity in receptor usage by T-cell-tropic feline leukemia viruses: Implications for the in vivo tropism of immunodeficiency-inducing variants. J. Virol..

[15] Chiu E.S., Hoover E.A., VandeWoude S. (2018). A retrospective examination of feline leukemia subgroup characterization: Viral interference assays to deep sequencing. Viruses.

[16] Roca A.L., Pecon-Slattery J., O’Brien S.J. (2004). Genomically intact endogenous feline leukemia viruses of recent origin. J. Virol..

[17] Szilasi A., Dénes L., Jakab C., Erdélyi I., Resende T., Vannucci F., Csomor J., Mándoki M., Balka G. (2020). In situ hybridization of feline leukemia virus in a primary neural B-cell lymphoma. J. Vet. Diagn. Investig..

[18] Hofmann-Lehmann R., Cattori V., Tandon R., Boretti F.S., Meli M.L., Riond B., Pepin A.C., Willi B., Ossent P., Lutz H. (2007). Vaccination against the feline leukaemia virus: Outcome and response categories and long-term follow-up. Vaccine.

[19] Miyake A., Watanabe S., Hiratsuka T., Ito J., Ngo M.H., Makundi I., Kawasaki J., Endo Y., Tsujimoto H., Nishigaki K. (2016). Novel feline leukemia virus interference group based on the env gene. J. Virol..

[20] Polani S., Roca A.L., Rosensteel B.B., Kolokotronis S.-O., Bar-Gal G.K. (2010). Evolutionary dynamics of endogenous feline leukemia virus proliferation among species of the domestic cat lineage. Virology.

[21] Chomczynski P., Sacchi N. (1987). Single-step method of RNA isolation by acid guanidinium thiocyanate-phenol-chloroform extraction. Anal. Biochem..

[22] Herring E., Troy G., Toth T., Forrester S., Weigt L., Herring I. (2001). Detection of feline leukaemia virus in blood and bone marrow of cats with varying suspicion of latent infection. J. Feline Med. Surg..

[23] Simons F.A., Vennema H., Rofina J.E., Pol J.M., Horzinek M.C., Rottier P.J., Egberink H.F. (2005). A mRNA PCR for the diagnosis of feline infectious peritonitis. J. Virol. Methods.

[24] Buonavoglia C., Martella V., Pratelli A., Tempesta M., Cavalli A., Buonavoglia D., Bozzo G., Elia G., Decaro N., Carmichael L. (2001). Evidence for evolution of canine parvovirus type 2 in Italy. J. Gen. Virol..

[25] Koç B.T., Oğuzoğlu T.Ç. (2020). A phylogenetic study of Feline Immunodeficiency Virus (FIV) among domestic cats in Turkey. Comp. Immunol. Microbiol. Infect. Dis..

[26] Adıgüzel E., Erdem-Şahinkesen E., Koç B.T., Demirden C., Oğuzoğlu T.Ç. (2023). The detection and full genomic characterization of domestic cat Orthohepadnaviruses from Türkiye. Vet. Med. Sci..

[27] Hall T.A. (1999). BioEdit: A User-Friendly Biological Sequence Alignment Editor and Analysis Program for Windows 95/98/NT. Nucleic Acids Symp. Ser..

[28] Kumar S., Stecher G., Li M., Knyaz C., Tamura K. (2018). MEGA X: Molecular evolutionary genetics analysis across computing platforms. Mol. Biol. Evol..

[29] Koç B., Oğuzoğlu T. (2023). Does endogenous feline leukemia virus occur as a risk factor?: A molecular characterization study from Türkiye: Molecular analysis of enFeLVs. J. Hell. Vet. Med. Soc..

[30] De Almeida N.R., Danelli M.G., da Silva L.H., Hagiwara M.K., Mazur C. (2012). Prevalence of feline leukemia virus infection in domestic cats in Rio de Janeiro. J. Feline Med. Surg..

[31] Studer N., Lutz H., Saegerman C., Gönczi E., Meli M.L., Boo G., Hartmann K., Hosie M.J., Moestl K., Tasker S. (2019). Pan-European study on the prevalence of the feline leukaemia virus infection–reported by the European Advisory Board on Cat Diseases (ABCD Europe). Viruses.

[32] Boeke J.D., Stoye J.P. (2011). Retrotransposons, endogenous retroviruses, and the evolution of retroelements. Sci. Rep..

[33] Powers J.A., Chiu E.S., Kraberger S.J., Roelke-Parker M., Lowery I., Erbeck K., Troyer R., Carver S., VandeWoude S. (2018). Feline leukemia virus (FeLV) disease outcomes in a domestic cat breeding colony: Relationship to endogenous FeLV and other chronic viral infections. J. Virol..

[34] Day M.J., Horzinek M., Schultz R., Squires R. (2016). WSAVA Guidelines for the vaccination of dogs and cats. J. Small Anim. Pract..

[35] Helfer-Hungerbuehler A.K., Spiri A.M., Riond B., Grest P., Boretti F.S., Hofmann-Lehmann R. (2015). No benefit of therapeutic vaccination in clinically healthy cats persistently infected with feline leukemia virus. Vaccine.

